# Akt promotes Endocardial-Mesenchyme Transition

**DOI:** 10.1186/2040-2384-1-2

**Published:** 2009-09-21

**Authors:** Kafi N Meadows, Seema Iyer, Mark V Stevens, Duanning Wang, Sharon Shechter, Carole Perruzzi, Todd D Camenisch, Laura E Benjamin

**Affiliations:** 1 Department of Pathology, Beth Israel Deaconess Medical Center, Harvard Medical School, Boston, MA, USA; 2 Department of Pharmacology and Toxicology, College of Pharmacy, The University of Arizona, Tucson, Arizona, USA

## Abstract

Endothelial to mesenchyme transition (EndMT) can be observed during the formation of endocardial cushions from the endocardium, the endothelial lining of the atrioventricular canal (AVC), of the developing heart at embryonic day 9.5 (E9.5). Many regulators of the process have been identified; however, the mechanisms driving the initial commitment decision of endothelial cells to EndMT have been difficult to separate from processes required for mesenchymal proliferation and migration. We have several lines of evidence that suggest a central role for Akt signaling in committing endothelial cells to enter EndMT. Akt1 mRNA was restricted to the endocardium of endocardial cushions while they were forming. The PI3K/Akt signaling pathway is necessary for mesenchyme outgrowth, as sprouting was inhibited in AVC explant cultures treated with the PI3K inhibitor LY294002. Furthermore, endothelial marker, VE-cadherin, was downregulated and mesenchyme markers, N-cadherin and Snail, were induced in response to expression of a constitutively active form of Akt1 (myrAkt1) in endothelial cells. Finally, we isolated the function of Akt1 signaling in the commitment to the transition using a transgenic model where myrAkt1 was pulsed only in endocardial cells and turned off after EndMT initiation. In this way, we determined that increased Akt signaling in the endocardium drives EndMT and discounted its other functions in cushion mesenchymal cells.

## Introduction

Prior to EndMT, the heart is a tube consisting of an inner endocardium and an outer myocardium separated by a thin layer of extracellular matrix called the cardiac jelly. On E9.5, signals from the myocardium and cardiac jelly induce a subset of endothelial cells in the endocardium to transform into mesenchyme cells. They migrate into the cardiac jelly and proliferate, eventually remodeling the cardiac cushions into heart valve leaflets and septa for a partitioned heart [1-4]. Several coordinated signals in the endocardium and myocardium that modulate EndMT in the AVC have been characterized. Developmental defects in cardiac tissues of TGFβ2 knockout mice, resulting in perinatal mortality, have been attributed to problems with epithelial-mesenchymal transition which underscores that increased TGFβ2 expression between E8.5 and E9.5 is necessary for EndMT to occur [5,6]. This induction is facilitated by endocardial activation of Notch which also stimulates Snail transcriptional repression of vascular endothelial (VE)-cadherin [7,8]. VE-cadherin becomes delocalized from cell junctions, facilitating the formation of the sprouting phenotype characteristic of mesenchyme cells [9]. VE-cadherin downregulation is a requisite for EndMT and, like EMT in epithelial cells, coincident N-cadherin upregulation marks the mesenchymal state. By E10.5, VEGF localization in the myocardium causes the cessation of EndMT [10,11]. It is unclear whether Akt mediates any of these signals, however, PI3K-Akt signaling has been established as a component of EndMT in a number of systems.

In mammary epithelial cells and tumors isolated from mouse mammary glands, Akt is activated in response to EMT induction [12,13]. The inhibition of PI3K-Akt signaling in metastatic breast tumor cells reduces EMT and transcriptional responses promoted by TGFβ [14]. Moreover, expression of myrAkt in squamous carcinoma cells is sufficient to drive EMT including the relocation of epithelial (E)-cadherin from cell junctions to cytoplasmic granules and the induction of mesenchyme markers, N-cadherin and vimentin [15]. Also, the importance of the Akt signaling pathway in EndMT is underscored by a number of downstream Akt pathways. Akt pathway targets including βCatenin, Notch, and Snail are essential for EndMT and regulate the formation of endocardial cushion [7,8,16]. Recently, PI3K signaling was determined to be necessary for AVC mesenchyme outgrowth, as pharmacological inhibition of Akt using an allosteric inhibitor (EMD Akt inhibitor XI) stunted mesenchyme outgrowth in cardiac cushion explants [17]. It is not clear from this pharmacological inhibition, which can target endocardial, myocardial and mesenchyme cells, whether initiation of EndMT is prevented or if there is a subsequent inhibition on the migration and proliferation of mesenchyme cells since the assay relies on counting the cells that have invaded the collagen gel. We have investigated the hypothesis that Akt signaling in endocardial cells initiates EndMT in the developing heart cushions.

## Materials and methods

### In Situ Hybridization

In situ hybridization was performed on E10.5 CD-1 embryos as described in [18]. Specific riboprobes designed to the 3' untranslated regions (3'UTRs) of Akt1 sense, 5' agactctgatcatcatccctgggt 3', and antisense, 5' actctcgctgatccacatcctgag 3' were produced using the T3 transcription reaction kit and Digoxigenin (DIG)-labeling (Roche).

### Immunohistochemistry

Timed matings between VE-cadherin:tTA and either TET:myrAkt1 mice or VE-cadherin:tTA and TET:lacZ were used to generate embryos expressing VE-cadherin:tTA, TET:myrAkt1, VE-cadherin:tTA/TET:myrAkt1, TET:lacZ, and VE-cadherin:tTA/TET:lacZ. E11.5 embryos were fixed in 3.7% paraformaldehyde, embedded in OCT, and sectioned. MyrAkt embryos were stained with phospho-Akt1 (Upstate) to confirm transgene expression and localization. LacZ embryos were stained with x-gal (Specialty Media) to confirm LacZ localization and were counterstained with eosin.

### Ex Vivo AVC Assay

The AVC explants were cultured as previously described in [19]. Relative differences in mesenchyme outgrowth were scored after 48 hours in culture (1 = monolayer growth, 2 = mesenchyme sprouts, 3 = mesenchyme sprouts with extensive migration). Inhibitor used: 10 μM LY294002 (Sigma). Antibody: Cy3 conjugated α SMA (Sigma).

### Isolation of primary mouse endothelial cells, Immunofluorescence, Western Blot and RT-PCR

Primary endothelial cells were isolated from the hearts or lungs of wildtype or VE-cadherin:tTA/TET:myrAkt1 mice as described previously [20]. VE-cadherin:tTA/TET:myrAkt1 cells were cultured in the presence of 2 μg/ml TET (Fischer) to suppress myrAkt expression. Immunofluorescent staining and western blot analysis were performed according to standard protocols. Antibodies: N-cadherin and VE-cadherin (BD-Biosciences), Snail (Abcam), Tubulin (Calbiochem). RT-PCR analysis was performed as described previously [21]. Primers: VE-cadherin sense, 5' ggccctggacagactgca 3' and antisense, 5' ttcgtggaggagctgatc 3'. GAPDH sense, 5' ggcaaattcaacggcacagt 3' and antisense, 5' aagatggtgatgggcttccc 3'.

## Results and Discussion

**Figure 1:**
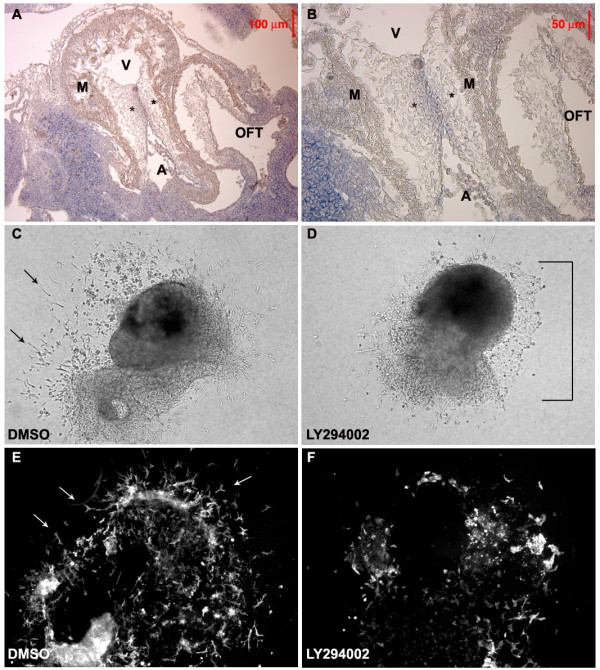
**Akt promotes EndMT during endocardial cushion formation**. (A and B) E10.5 Akt1 mRNA is expressed in the endocardial cushion (*) of the AVC, but not in the myocardium (M) of the ventricle (V), atria (A) or outflow track (OFT). The image was taken under 4× (A) and 10× magnification (B). (C-F) AVC Explants were treated with DMSO (C and E) or pharmacological inhibitor of PI3K (LY294002) (D and F). The mesenchyme outgrowth (arrows) detected in explants was significantly reduced in explants treated with L294003, both in the brightfield images (C and D) DMSO treated as well as (E and F) α SMA stained fluorescent images. What outgrowth there was with LY294002 was the cobblestone morphology of an endothelial monolayer (D, note bracket). These images were representative of multiple explants (n>10) in independent experiments.

We analyzed the expression pattern of Akt mRNA in normal embryos by in-situ hybridization. All three Akt isoforms are expressed in the heart during the developmental window where endocardial cushions are formed [22]. While Akt2 and Akt3 expression were more widespread in all cells of the cardiac cushion (not shown), we found Akt1 mRNA localized strictly to the endocardial layer of E10.5 heart cushions (Figure 1A and 1B) suggesting that Akt1 may have a unique role in EndMT. To examine the role of Akt in EndMT, we utilized an AVC explant assay, which recapitulates EndMT observed in vivo. The invading cells which grow out from AVCs explanted onto 3-dimensional collagen are similar to the cushion cells of the embryonic heart [23], and the time course of the growth in this ex vivo culture recapitulates the onset of EMT that occurs in vivo [24]. Control (DMSO-treated) had numerous elongated cells sprouting into the collagen gel at the periphery of the explant after 48 hours in culture (Figure 1C), while AVC explants treated with the PI3K inhibitor LY294002, had stunted mesenchyme outgrowth, which was replaced by a cell monolayer with a cobblestone morphology indicative of endothelial cell growth (Figure 1D). We detected a dramatic reduction in α-SMA positive mesenchymal cells in LY294002 treated explants (Figure 1F) compared to controls (Figure 1E).

**Figure 2:**
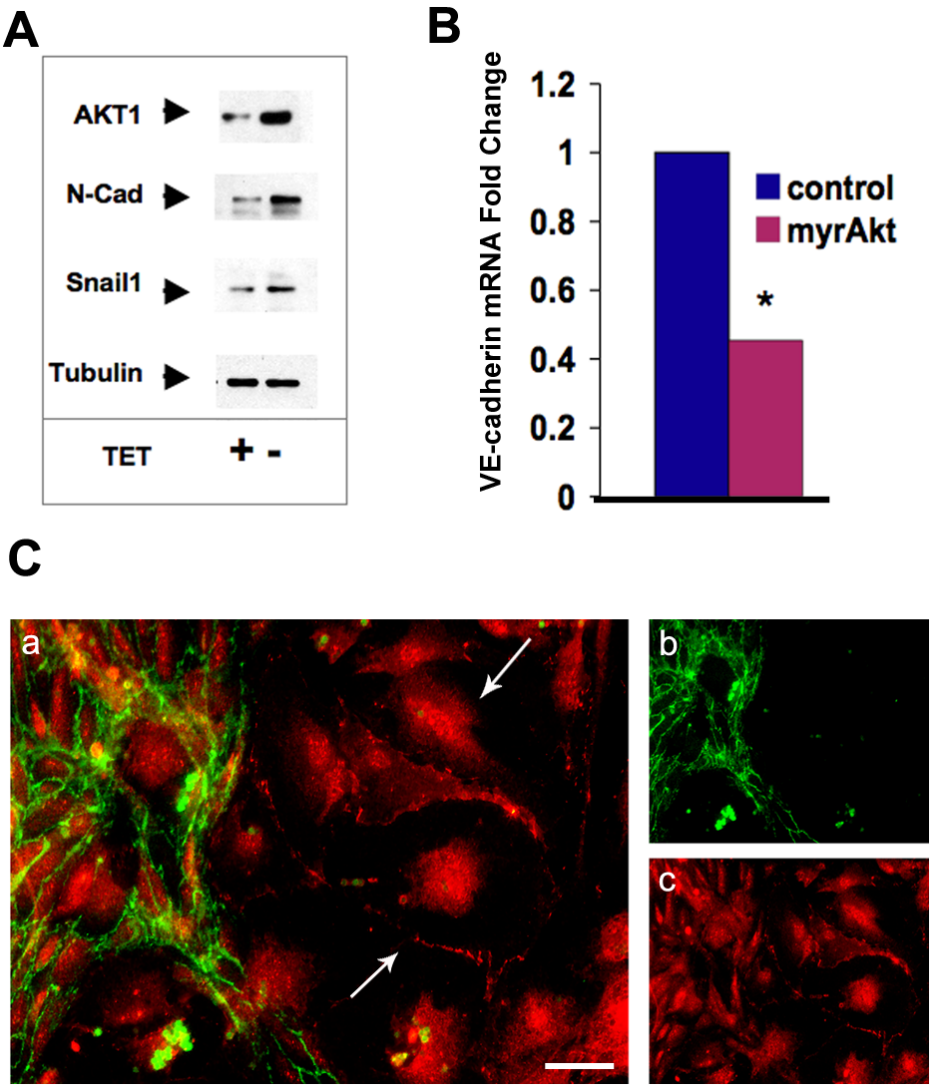
**EndMT markers were induced in VE-cadherin:tTA/TET:myrAkt1 Endothelial cells**. (A) Protein lysates were harvested from VE-cadherin:tTA/TET:myrAkt1 endothelial cells and were treated with or without TET for 72 hours. In the presence of TET myrAkt1 is suppressed, while induction occurs in the absence of TET. An increase in N-Cadherin and Snail1 was observed by Western Blot when myrAkt1 is expressed (-TET). (B) RT-PCR of RNA isolated from VE-cadherin:tTA/TET:myrAkt1 endothelial cells shows a significant decrease in VE-cadherin mRNA expression when myrAkt1 was expressed, *student T-test p-value = 0.015. (C) Endothelial cells cultured in the absence of tetracycline (increased myrAkt1) were stained with VE-cadherin (green, b) and N-cadherin (red, c). The merged images reveal populations of cells, which had lost VE-cadherin expression (a, arrows). These studies were representative of multiple experiments (n>2) in independent experiments.

Akt activation and Snail induction have been clearly demonstrated in a number of epithelial cell systems [12,25]. Snail acts as a transcriptional repressor of VE-cadherin when it is induced in endothelial cells during EndMT [8]. Loss of VE-cadherin and induction of N-cadherin are not only hallmarks of EndMT but likely facilitate loss of cell-cell adhesion and the onset of a migratory phenotype. We sought to determine whether Snail was induced in endocardial cells in response to elevated Akt1 signaling by isolating endothelial cells from neonatal mice with a myrAkt1 transgene driven by the VE-cadherin promoter [26]. This expression of myrAkt1 is repressible by tetracycline, so we used media with and without tetracycline to control Akt signaling allowing us to measure changes in gene expression in response to the increased Akt1 signal. When myrAkt1 was expressed, we observed increased Snail and N-cadherin protein (Figure 2A) but decreased VE-cadherin mRNA (Figure 2B), consistent with Snail's transcriptional repression of VE-cadherin. This observation links to our previous observation that GSK3β phosphorylation is increased in myrAkt endothelial cells [20]. Unphosphorylated GSK3β negatively regulates Snail by phosphorylating it at two consensus motifs, causing its nuclear export and degradation [27]. Although consistent, at this point we cannot be sure whether the phosphorylation and inactivation of GSK3β by myrAkt directly causes Snail induction or if Snail is regulated through an alternative pathway. Also consistent with these findings, when cultures were propagated in the absence of tetracycline, we not only had increased growth and survival of the cells, but also found that a population of VE-cadherin negative, N-cadherin positive cells emerged (Figure 2C). The VE-cadherin positive cells usually also contained N-cadherin, but the subpopulation that became VE-cadherin negative appeared by morphology to be less cobblestone and more motile. This is consistent with published reports that VE-cadherin expression in endocardial cells becomes downregulated once they become mesenchymal and invade the endocardial cushion [16]. These observations suggest that a subset of our cultures were indeed undergoing an EndMT-like transition in the presence of constitutive Akt1 signaling. Because we isolated endothelial cells from the whole heart, it is difficult to know whether this VE-cadherin negative subpopulation is derived from endocardium or endothelial cells from the vessels in the heart. There is a precedent for adult heart endothelial cells to undergo EndMT after injury leading to scarring and fibrosis [28]. Thus it may be that endothelial cells from blood vessels and not just endocardial cells are driven by Akt signaling to EndMT.

**Figure 3:**
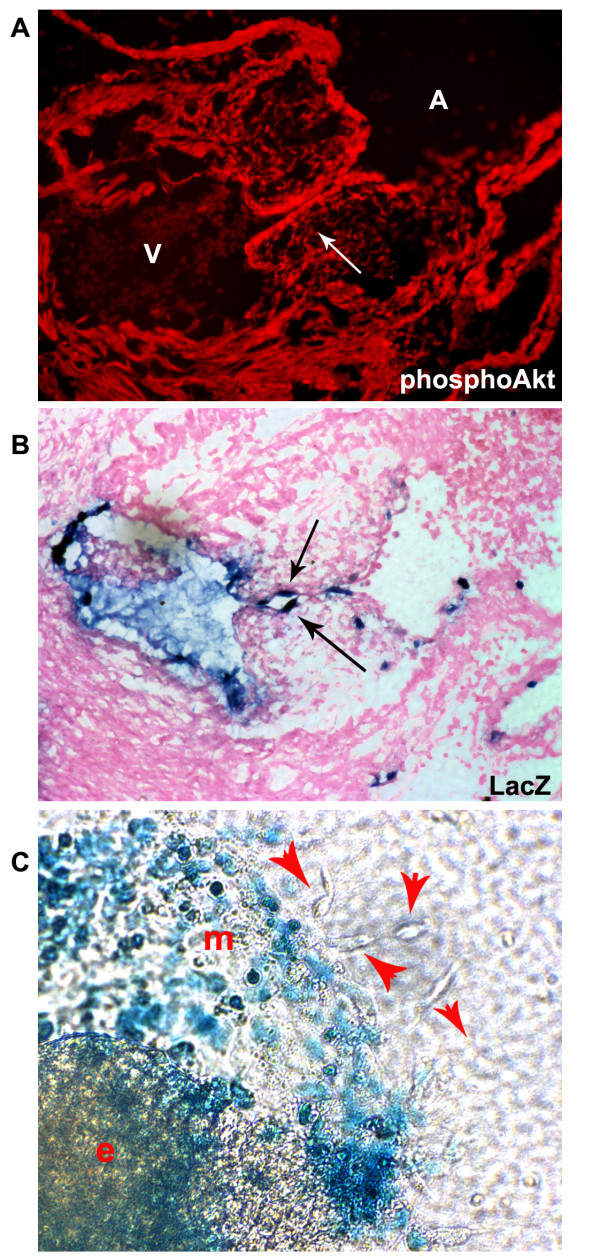
**The VE-cadherin-tTA transgene drives responder expression only in the endocardium, not the cardiac cushion mesenchyme**. (A) E11.5 VE-cadherin:tTA/TET:myrAkt embryos stained with phospho-Akt showed increased Akt activation in the endocardium. (B) LacZ staining of E11.5 VE-cadherin:tTA/TET:LacZ embryos confirmed the endothelial specificity of the VE-cadherin promoter. (C) In ex vivo heart cushion explants (e), endothelial layer monolayer (m) outgrowth expressed lacZ but the mesenchymal outgrowth cells (red arrowheads) did not, again confirming the VE-cadherin-tTA gene would not be expressed and thus not drive TET-promoter expression in the mesenchyme. The figures shown are representative images from n>5 double transgenic explants assessed. Each litter gives 25% double transgenic offspring.

Pharmacological inhibition in the AVC explants disrupts the PI3K/Akt signaling pathway in myocardial, endocardial, and mesenchyme cells. It is possible that Akt signaling regulates multiple stages of endocardial cushion formation including promoting survival, proliferation and migration of mesenchymal cells. While it was clear that the PI3K/Akt pathway is integral to EndMT, in part based on the expression pattern of Akt1 that we observed (Figure 1B), we hypothesized that the Akt1 signaling pathway acts in endocardial cells to promote the commitment decision to undergo EndMT before playing a role in mesenchymal cell biology. To investigate this we utilized our inducible system of conditionally expressing myrAkt driven by the VE-cadherin promoter in endothelial cells as described in Figure 2. We validated that we have increased Akt activity (pAkt) in the endocardial layer of the developing cardiac cushions (Figure 3A), though clearly we can see some Akt signaling in cushion mesenchyme and other embryonic tissues. Because EndMT leads to downregulation of the VE-cadherin promoter, our expectation was that VE-cadherin-driven myrAkt1 expression would be downregulated once the endocardium undergoes EndMT. To confirm this we examined hearts and heart explants from a TET:lacZ reporter responder line bred to the VE-cadherin-driven transcriptional activator (tTA) line. In these mice, the VE-cadherin-driven tTA gene drives endocardial expression of lacZ in the same location where it drives myrAkt1 when crossed to the TET-myrAkt1 mouse. We detected lacZ staining only in endocardial cells of the developing cushions but not in cushion mesenchyme (Figure 3B). Consistent with these in vivo observations, ex vivo explants showed lacZ staining in many of the cobblestone endocardial cells growing out of the explant, but the mesenchymal cells were lacZ negative (Figure 3C). These data confirmed that myrAkt1 was expressed in the endocardium and turned off during mesenchymal outgrowth.

**Figure 4:**
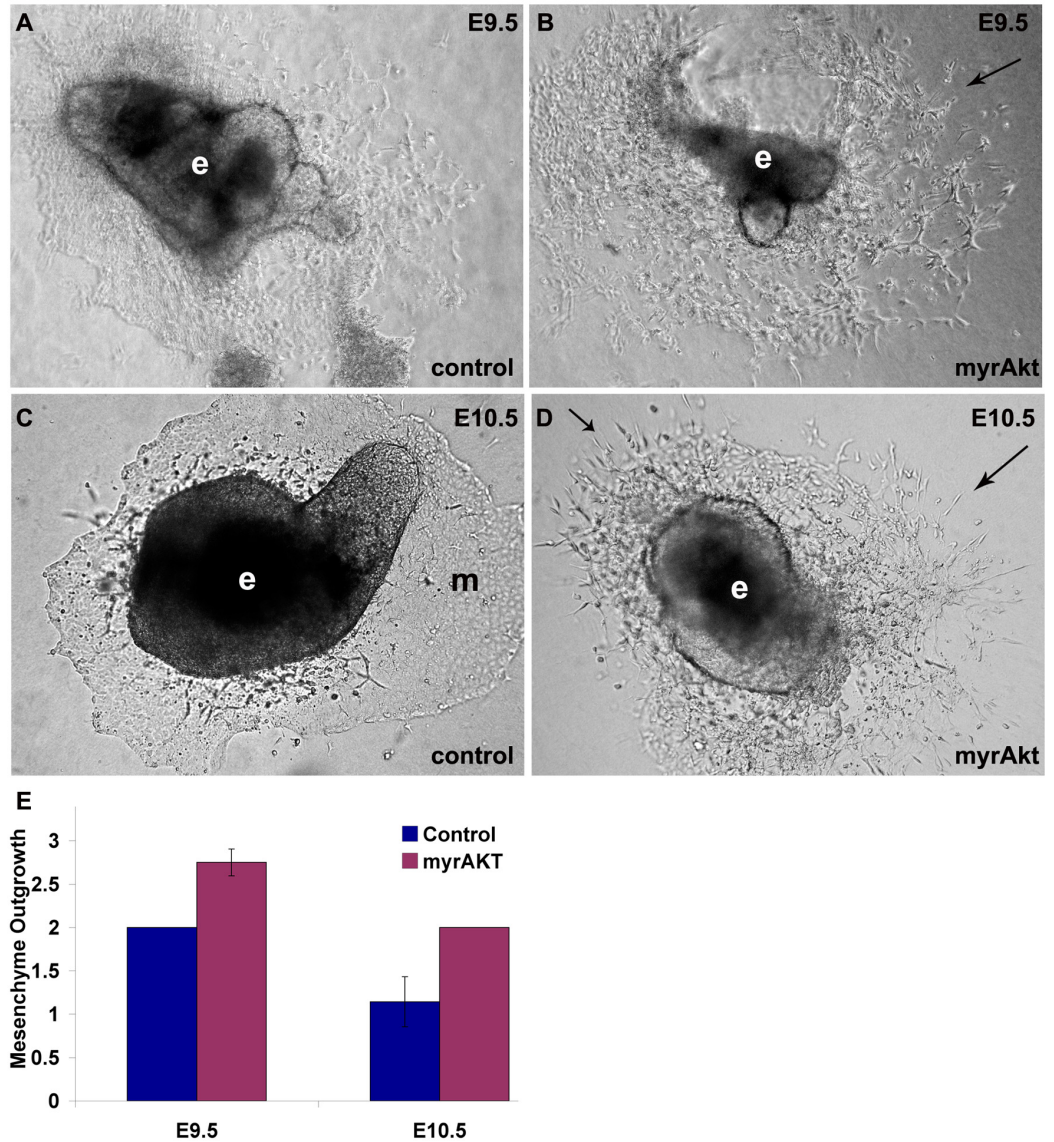
**myrAkt promotes mesenchyme outgrowth**. (A-D) AVC explants (e) cultured on 3-D collagen gels had increased mesenchyme outgrowth of E9.5 VE-cadherin:tTA/TET:myrAkt AVCs (arrows) (B) when compared to control (A). This was more pronounced in E10.5 VE-cadherin:tTA/TET:myrAkt AVCs explants (D), where explants were still sprouting mesenchyme (arrows), while outgrowth of the control AVC explants was composed of an endothelial monolayer (m) (C). (E) Scoring of relative differences in mesenchymal invasion showed an increase in mesenchyme outgrowth of VE-cadherin:tTA/TET:myrAkt AVCs at E9.5 and E10.5 compared to control explants. The figures shown are representative images from n>5 double transgenic explants assessed. Each litter gives 25% double transgenic offspring.

To test whether we could drive EndMT by increasing Akt1 signaling only in the endocardium, we harvested the hearts from double transgenic mice with the VE-cadherin promoter driving myrAkt expression, bisected them and placed them onto collagen gels to allow mesenchymal outgrowth and invasion into the gel. Sustained activation of myrAkt in all endothelial cells leads to embryonic lethality from vascular malformations and edema [26]. To circumvent this problem, we kept the pregnant mothers on tetracycline until E8.5, just before induction of EndMT in the AVC, and isolated embryos at E9.5 & E10.5. When the AVC endocardial cushions from E9.5 myrAkt mice were explanted onto collagen gel, we observed more invasive mesenchyme outgrowth (Figure 4B) than the control explants (Figure 4A). By E10.5 the AVC has already undergone EndMT and lost the potential to induce collagen gel mesenchymal invasion. Control AVCs at this later stage show decreased EndMT resulting in a more cobblestone endothelial monolayer (Figure 4C). However, mesenchyme outgrowth potential was maintained in explants from myrAkt AVCs (Figure 4D).

Since the tTA is turned off when the *ve-cadherin *promoter is downregulated in EndMT, we interpret these data to demonstrate that myrAkt expression in advance of EndMT is sufficient to drive increased EndMT in the endocardium. Together, these data establish a role for Akt1 as a novel signaling component driving the endocardial commitment to EndMT in the developing heart. Interestingly, deletion of only Akt1 does not impair mesenchyme transition, as embryos from Akt1 null mice develop normally. It is likely Akt3 compensates for the absence of Akt1 when it is disrupted, as it is upregulated in endothelial cells isolated from Akt1 knockout endothelial cells [29]. Furthermore, while disruption of no single Akt gene is lethal, Akt1/Akt3 null embryos die between E11 and E12, and have vascular defects and hemorrhages, implicating an endothelial defect. Furthermore, a requirement for Akt1 and Akt3 in heart function was demonstrated by the observation that postnatal day 3 (P3) hearts from Akt1^-/- ^Akt3^+/- ^mice are reduced in size and have enlarged atria and ventricles indicative of a failing cardiovascular system [22]. These mice were reported to have septation defects and thickened valves, which implicates defects in endocardial cushion development and maturation. Together, these data suggest that Akt1 can drive EndMT, and there can be functional redundancy in the Akt isoforms in the absence of Akt1.

Recent studies indicating that endocardium undergoes EndMT following heart injury and leads to fibrosis open the possibility that Akt1 signaling may also participate in pathological processes in adult hearts [28]. Further study is required to investigate this possibility and to fully elucidate the signaling cascade driving Akt-mediated EndMT. In addition, the unique contribution Akt has in the myocardial and mesenchyme cell populations during endocardial cushion formation need to be determined. Our study defines for the first time a novel role for Akt1 at the earliest stages of endocardial commitment to becoming a mesenchymal phenotype.

## Competing interests

The authors declare that they have no competing interests.

## Authors' contributions

KNM carried out the ex vivo atrioventricular canal (AVC), immunohistochemistry, immunofluorescence, and western blot studies, and drafted the manuscript. SI participated in the manuscript revisions and submission and intellectual input. MS carried out in situ hybridization studies. DW carried out in situ hybridization studies. SS conducted the real-time polymerase chain reaction (RT-PCR) experiments. CP isolated the primary mouse endothelial cells and conducted immunofluorescence experiments. TDC was the collaborating principle investigator overseeing MS and DW for the in situ studies. LEB was the primary principle investigator who participated in the study conception, funding, execution, analysis, and manuscript preparation. All authors read and approved the final manuscript.
